# Biomaterials research of China from 2013 to 2017 based on bibliometrics and visualization analysis

**DOI:** 10.7717/peerj.6859

**Published:** 2019-05-06

**Authors:** Dandan Hou, Xuewei Bi, Zhinan Mao, Yubo Fan, Xiangming Hu, Xiaoming Li

**Affiliations:** 1 Key Laboratory for Biomechanics and Mechanobiology of Ministry of Education, School of Biological Science and Medical Engineering, Beihang University, Beijing, China; 2 Beijing Advanced Innovation Center for Biomedical Engineering, Beihang University, Beijing, China; 3 School of Humanities and Social Sciences, Beihang University, Beijing, China; 4 School of Materials Science and Engineering, Beihang University, Beijing, China

**Keywords:** Biomaterials, Bibliometrics, Visualization, General information, Research trends

## Abstract

**Objectives:**

This study aims to evaluate the changes of development trends and research hotspots of biomaterials research from 2013 to 2017, which can identify the general information of papers and explore the changes of research content, thus providing perspectives for the development of biomaterials in China and other countries.

**Methods:**

Data of the paper were retrieved from the Web of Science Core Collection, and then analyzed by the bibliometric and CiteSpace visualization analysis.

**Results:**

It was found that a total of 3,839 related papers had been published from the year 2013 to 2017. The analysis of the articles showed that the annual quantity and quality of the articles in the biomaterials research have been increasing since 2013, and the Wang L / Chinese Academy of Sciences were the most productive author/institution. Meanwhile, the keywords “in vitro”, “scaffold”, “nanoparticle” , “mechanical property”, and “biocompatibility” have the relatively higher frequency, and the keywords “apatite”, “deposition”, and “surface modification” have the strongest burst citation.

**Conclusions:**

After statistics and analysis, we found that biomaterials is a promising research field. The study may be helpful in understanding research trends in this field.

## Introduction

The writing of papers is a necessary stage of scientific research. The quantity and quality of papers are important indicators reflecting their research achievements and scientific research strength (Gutierrez-Salcedo et al., 2018). According to the changes of scientific literature, the history, current status, and development trends of scientific research can be studied and analyzed. Bibliometrics can be defined as the combination of statistics and philology, which uses statistical methods to study the relationship between literature and information (Godin, 2006; Gimenez, Salinas & Manzano-Agugliaro, 2018). The application of bibliometric methods has been very extensive, which can reveal the law of variation of the amount of papers, analyze the process of the development about the discipline, and evaluate the research level of different countries, regions, and organizations in specific disciplines at the macro levels (Li et al., 2018; Zhao, 2018). Meanwhile, bibliometrics assists researchers in obtaining a large amount of information, which has great application in the evaluation of scientific research performance (Ding et al., 2013; Miao, Zhang & Yin, 2018).

Biomaterials from various origins have been used for the repair of human tissues and organs (Garg & Goyal, 2014; Hinderer, Brauchle & Schenke-Layland, 2015). They have evolved from bio-inert (Jones & Hench, 2003), bio-active (Shirtliff & Hench, 2003), and controlled degradation materials (Nair & Laurencin, 2007) to functionalized materials (Ossipov, 2015), which not only have good biological properties, but also stimulate specific cell responses and induce tissue regeneration (Li et al., 2012; Hong et al., 2011; Fazal & Latief, 2018). With the development of biology and materials science, biomaterials are developing at an astonishing rate. Nanotechnology (Qian et al., 2017; Huang et al., 2018; Li et al., 2016a; Li et al., 2016b), surface modification technology (Chen et al., 2018; Ren et al., 2015; Ai et al., 2017; Wang et al., 2017), 3D printing (Kelly et al., 2018; Zhu et al., 2016), stem cell technology (Gaffney, Wrona & Freytes, 2018; Merceron et al., 2008) and other leading-edge technologies are accelerating the innovation of biomaterials (Dalby et al., 2004; Li et al., 2016a; Li et al., 2016b; Zhang et al., 2018). The government of China has been increased the investment in research and development, and continued to issue the policies to provide guidance for the development of biomaterials. During the period of “12th Five-Year Plan”, a series of policies were introduced to promote the development of biomaterials. “The 12th Five- Year Development Plan for New Materials Industry” promulgated by the Ministry of Industry and Information Technology clearly stated that the research on biomaterials must be improved to increase the biocompatibility and chemical stability of biomaterials, while the biomaterials with high performance and low cost can be developed. The “China Manufacturing 2025” plan issued by the State Council stated that the preparation and development of strategic frontier materials such as biomaterials should be put in the preferential position. In terms of government input, 400 million yuan was invested in biomaterials research during the “11th Five-Year” period. During the “12th Five- Year Plan” period, it had increased to 510 million yuan, and 100 billion yuan during the “13th Five-Year Plan”. In this paper, we studied the characteristics of papers and assess research topics, emerging trends and frontiers in the biomaterials research of China by using the methods of bibliometric and visualization analysis.

## Materials & Methods

### Data collection

The Web of Science Core Collection was selected as the source of data retrieval, which is an important database platform for domestic and foreign scholars to retrieve and obtain relevant academic literature information (Wang, Fang & Sun, 2016). The search theme was set as biomaterials, the time range was set from 2013–2017, and the country was limited to China. The total number of papers for each year and the number of different types of papers were collected and recorded in Excel. Based on the number of different paper types collected each year, the article (a type of papers) was selected for the subsequent analysis. Then the number of articles per year, and the annual impact factor of the journals in which the articles were published were all collected and recorded in Excel. Journal impact factor was obtained from the Journal Citation Analysis Database (Journal Citation Report, JCR).

### Statistical methods

The performing statistical analysis and visualizing analysis for all articles were analyzed by the ORIGIN2017 and CiteSpace (5.1.R8SE). ORIGIN2017 was used to describe the trends of the quantity and quality of articles, while CiteSpace was used to analyze the collaboration between authors/institutions, and identify the research topics, emerging trends and frontiers in the biomaterials study.

## Results

### Basic analysis of papers from 2013–2017

#### Quantitative analysis

**Figure 1:**
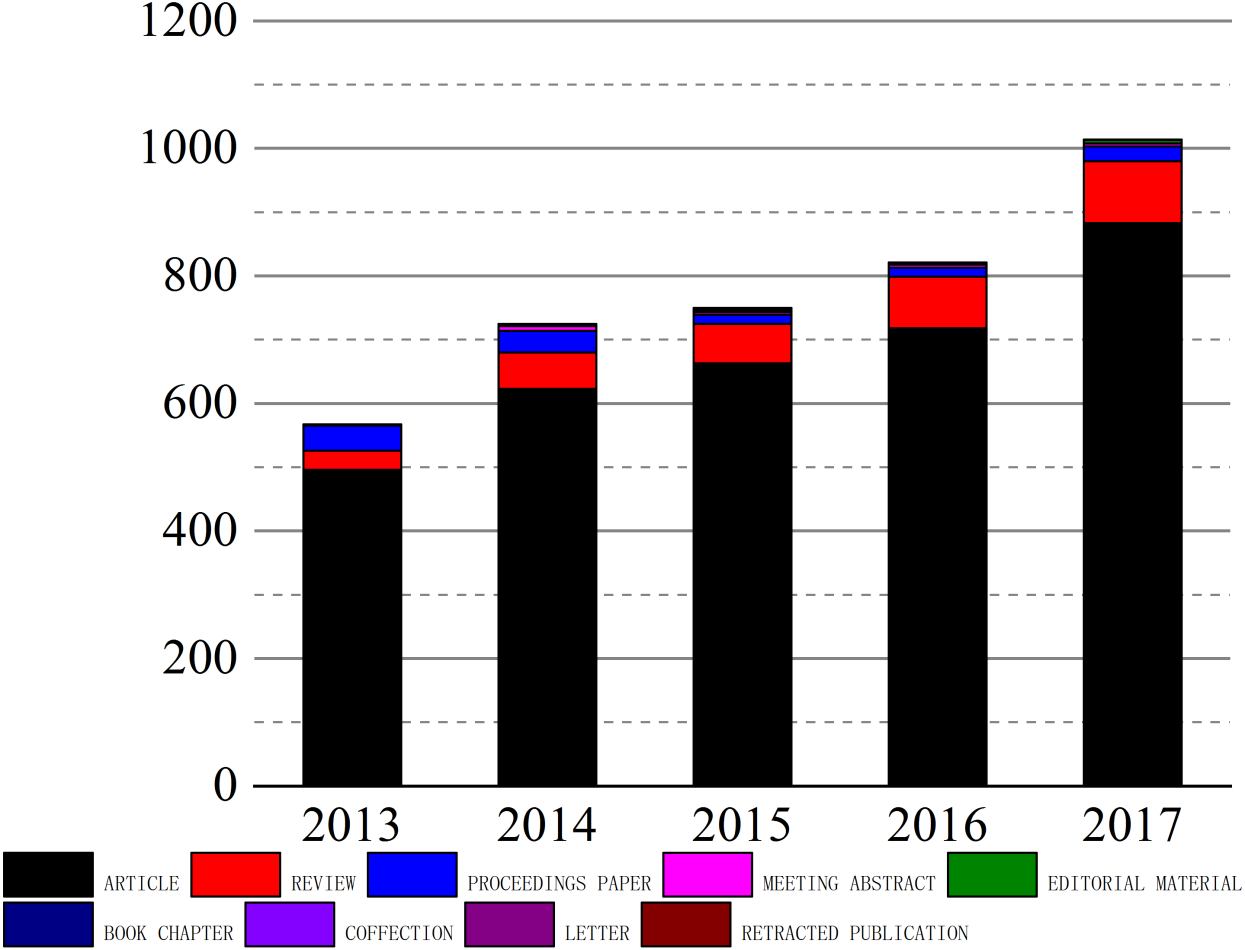
Number and types of papers published from 2013 to 2017. The figure shows the general condition of papers in the field of biomaterials of China from 2013 to 2017.

The amount of papers can intuitively reflect the output of a given field over the years, and show the trends of development in a certain area (Durieux & Gevenois, 2010). Figure 1 shows the general condition of papers in the field of biomaterials of China from 2013 to 2017. The total number of published papers was 3989. Among them, there are 1005, 815, 746, 713, and 560 papers in 2017, 2016, 2015, 2014, and 2013 (Dataset S1). In five years from 2013 to 2017, the number of papers has increased steadily as the papers in 2017 increased by 89.1% over 2013. In addition, there are 9 various types of papers published in China in the field of biomaterials, during the study period, and the largest number of papers are articles (3383), accounting for 88.12%, followed by reviews (327), accounting for 8.5%, the number of editorial material and meeting abstract is 21 and 15, and accounting for 0.54% and 0.39%. The book chapter, letter, retracted publication, and the correction occupied a tiny position. Articles are the largest proportion of annual papers, and they are selected for further analysis in view of the effectiveness of the papers.

#### Quality analysis

**Figure 2:**
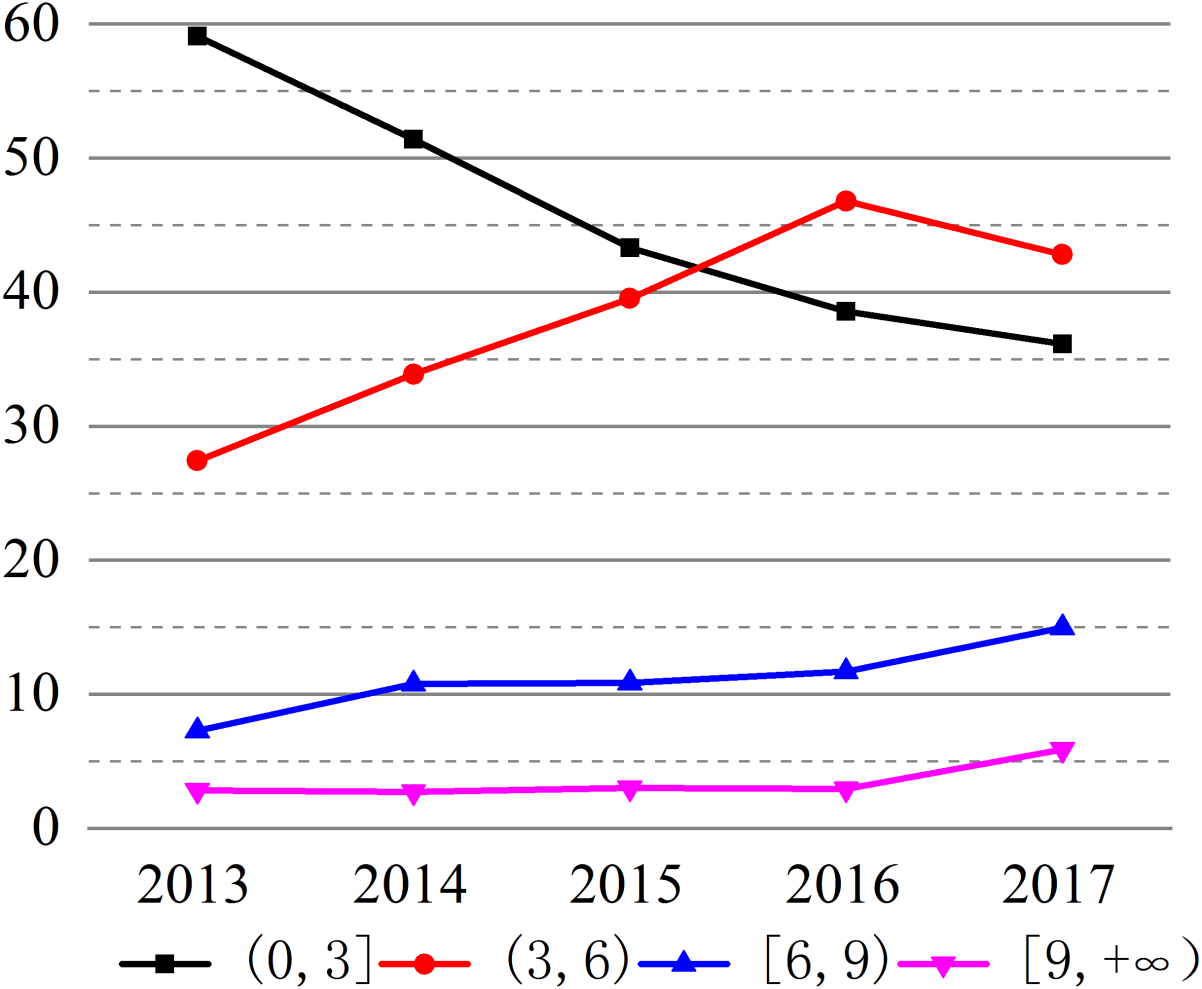
Trends of impact factors of articles from 2013 to 2017. The figure shows the changes of impact factors of journals published articles from 2013 to 2017.

**Figure 3:**
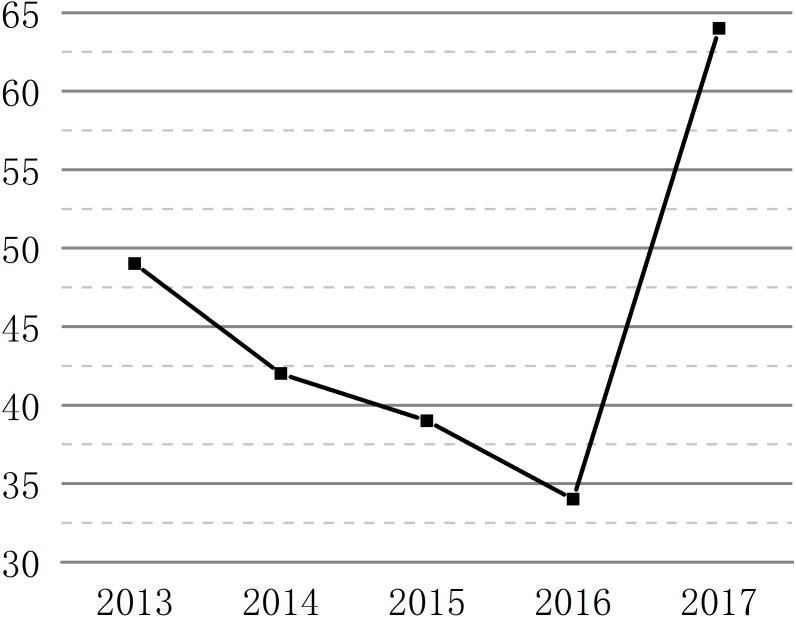
h-index of articles from 2013 to 2017. The figure shows the changes of h-index of published articles from 2013 to 2017.

Scientists tend to publish their research works in the journals with high impact factor. The articles in high impact journals is also widely believed to be capable of representing the high quality of the research (Rowlands & Nicholas, 2006). From 2013 to 2017, 563 journals have published the articles in biomaterials research, the number of journals involved every year is 192, 211, 219, 227, and 265 in 2013, 2014, 2015, 2016, and 2017 respectively (Datasets S2–S6), indicating that the field of biomaterials research continues to expand. The changes of impact factors of journals published articles from 2013 to 2017 are shown in Table 1 and Fig. 2. The total and average impact factors of journals published articles continue to increase from 2013 to 2017. The number of articles that impact factors below 3.0 is decreasing from 2013 to 2017, reducing from 59.1% (2013) to 36.12% (2017). There are few articles with impact factors over 9.0, which about 124 of the total articles, accounting for 3.66%, 14 in 2013, 17 in 2014, 20 in 2015, 21 in 2016, and 52 in 2017 (Datasets S7–S11). From the quality of the articles, the number of low impact factor articles is gradually decreasing and the number of high impact factors is gradually increasing. The h-index is another index used to evaluate the quality of articles (Costas & Bordons, 2007), as can be seen from the Fig. 3, the highest h-index in 2017 is 64, which shows that 64 high-quality academic articles were published in China in 2017, and the citation frequency of these articles is no less than 64. The other indexes are 49 in 2013, 39 in 2014, 39 in 2015, 34 in 2016.The h-index is slightly lower between 2013 and 2016, which maybe related to the cited frequency that is affected by time, this is also highlights the extraordinary progress of the quality of China’s publications in the field of biomaterials in 2017 compared with 2013.

**Table 1:** Impact factors of journals published articles from 2013 to 2017. The general information of impact factors of journals published articles from 2013 to 2017 is shown in Table 1.

Impact factor	2013	2014	2015	2016	2017
Total	560.41	615.63	687.37	773.85	984.90
Highest	36.43	17.49	18.96	21.70	21.95
Average	2.87	2.90	3.14	3.39	3.73

#### Authors and institutions analysis

**Figure 4:**
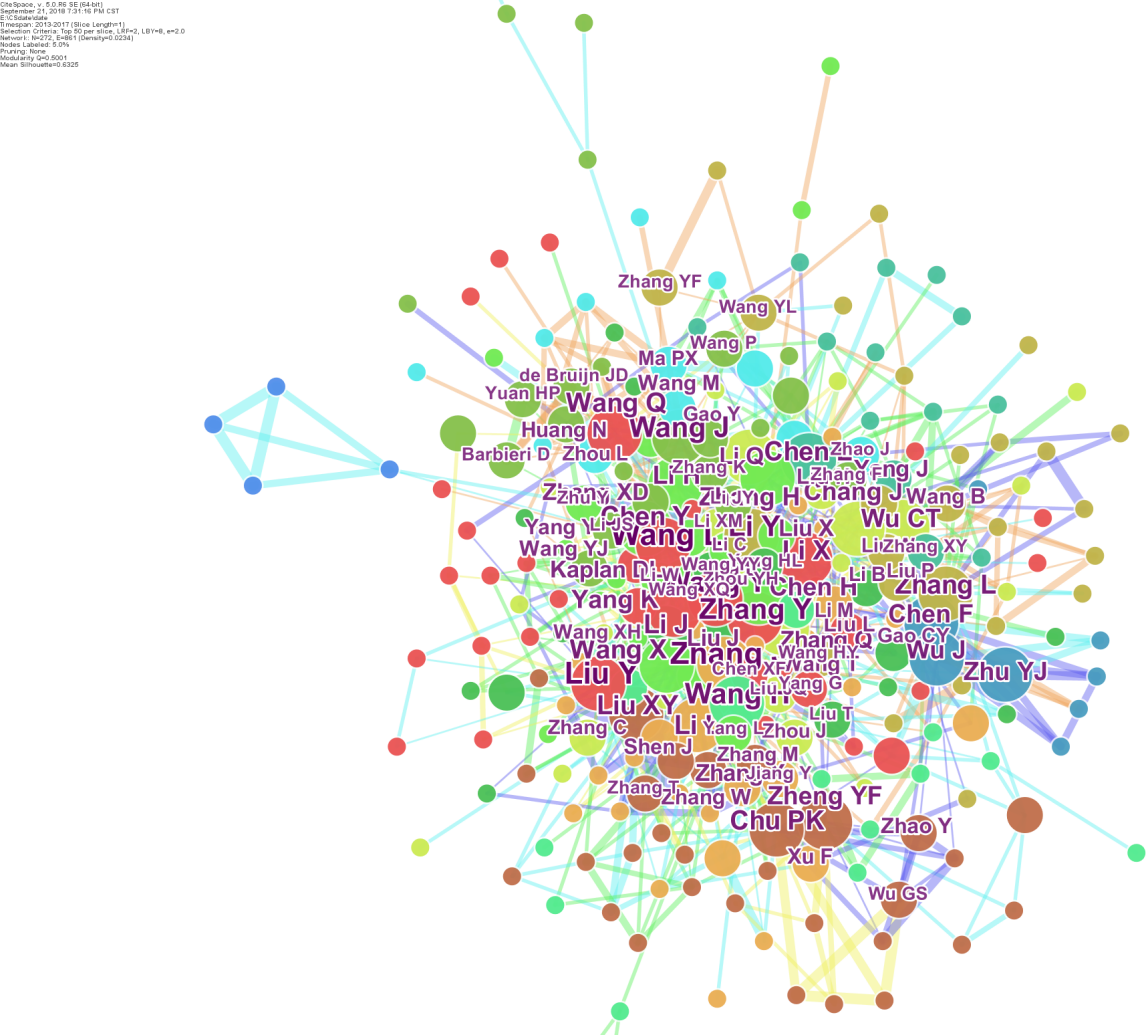
The knowledge mapping of authors. The knowledge mapping with the authors as the network node is shown. The size of each circle node represents the number of articles published by the author.

**Figure 5:**
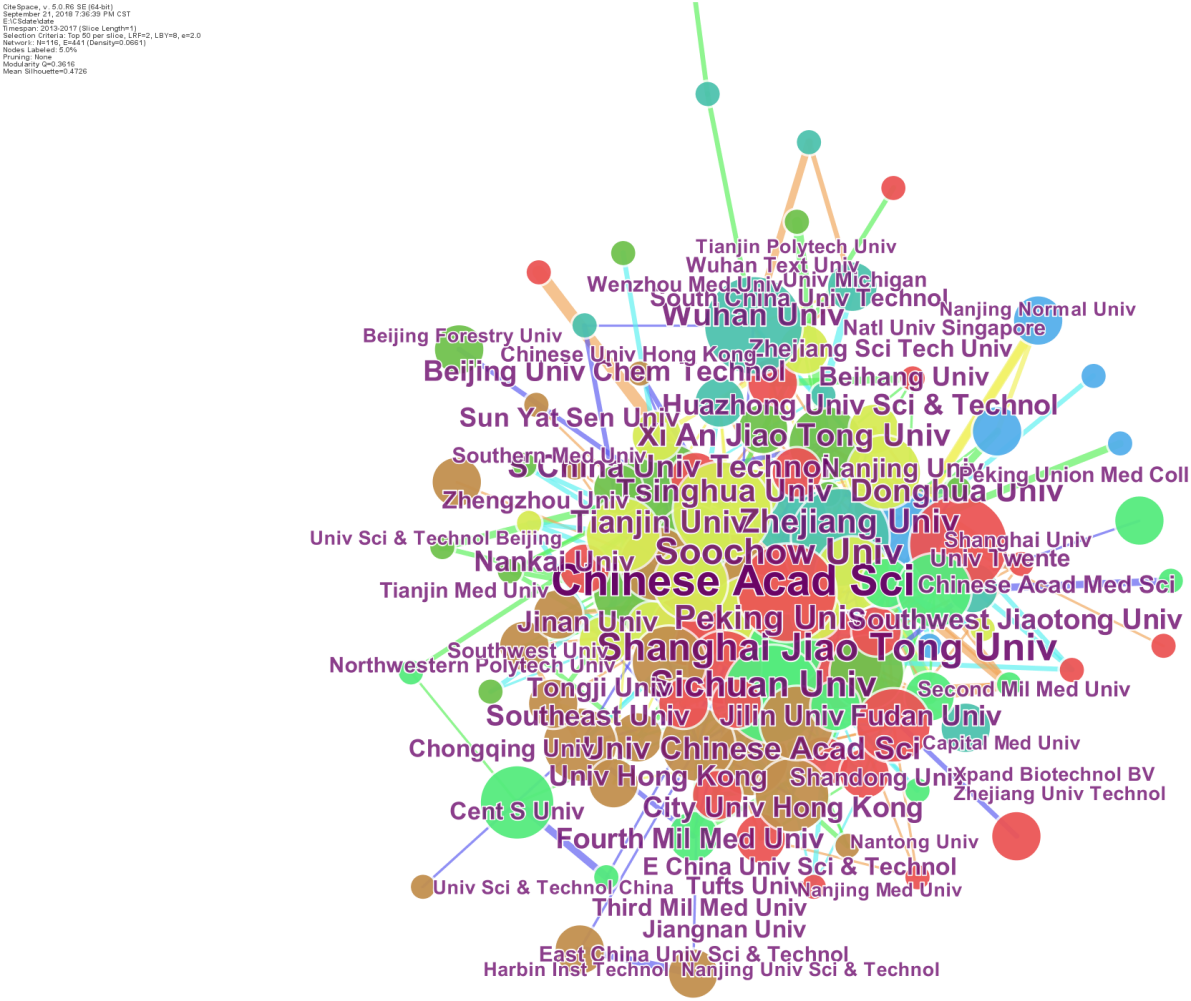
The knowledge mapping of cooperation institutions. The knowledge mapping with the institutions as the network node is shown. The size of each circle node represents the number of articles published by the organization.

The scientific collaboration has attracted wide attention of bibliometric researchers (Wallace, Lariviere & Gingras, 2012; Li et al., 2018). The data from the Web of Science is saved as a text document and then imports into Citespace software, while the time is set from 2013 to 2017. The knowledge mapping with the authors/institutions as the network node is drawing in Figs. 4 and 5. The size of each circle node represents the number of articles published by the author/organization. The larger circle node represents the greater number of articles. There are nine high- frequency authors (frequency ≥30) in this field, as shown in Table 2. Wang L appeared most frequently (51), followed by Liu Y (47), Zhang J (44), and Zhang Y (41) et al. In terms of institutions, as shown in Fig. 5, the Chinese Academy of Sciences (CAS) is the most contributive institution in biomaterials research with the highest frequency (268), followed by Shanghai Jiaotong University (146) and Sichuan University (118). Moreover, the knowledge mapping also shows a strong cooperative network that has been formed between researchers and institutions. It should be noted that the inter-institutional cooperation is mostly domestic institutions. For the organizations with the inter-institutional cooperation frequency greater than 10, only Tufts University, University of Twente, University of Michigan, and National University of Singapore are overseas institutions, revealing that international scientific and technological exchanges and cooperation are insufficient.

### Keywords analysis

#### Evolutions of research topic

**Figure 6:**
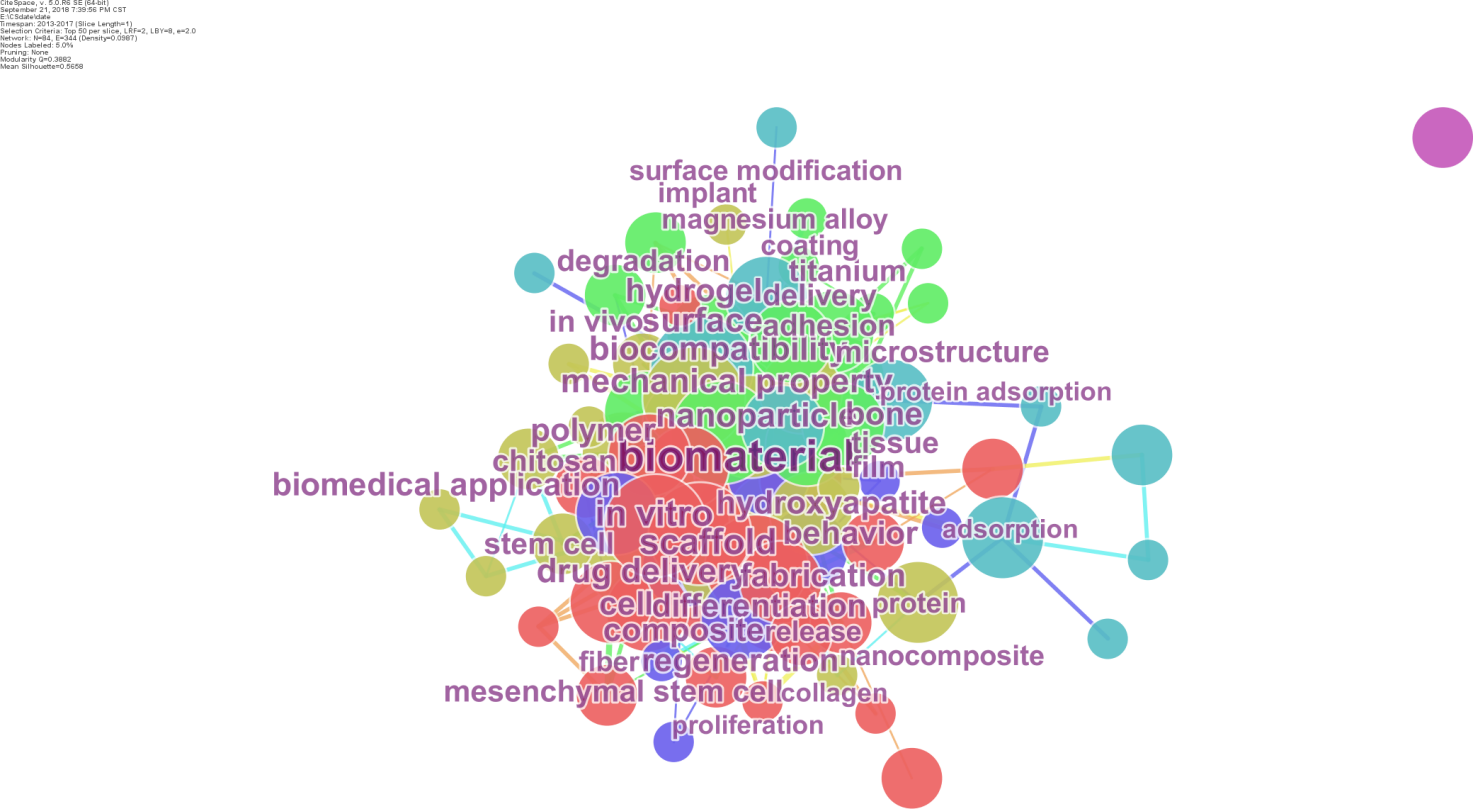
Co-occuring keyword network. The co-occuring keyword network with the frequency greater than 10 is shown. Keywords are the core representation of the research topic of academic articles, which can be used to discover changes in research hotspots, and reveal the intrinsic link of knowledge distribution in a certain subject area to some extent.

Keywords are the core representation of the research topic of academic articles, which can be used to discover changes in research hotspot, and reveal the intrinsic link of knowledge distribution in a certain subject area to some extent (Xiang, Wang & Liu, 2017; Khan & Wood, 2015). The co-occuring keyword network with the frequency greater than 10 was shown in Fig. 6. Among them, there are 57 keywords in 2013, which accounts for 67.8% of the total keywords, and 12 new keywords in 2014, five new keywords in 2015, 6 new keywords in 2016, five new keywords in 2017, which means the mainly research contents of biomaterials have not changed for a long time. The keywords such as “in vitro”, “scaffold”, “nanoparticle”, “mechanical property”, “biocompatibility”, “drug delivery”, “surface”, and “hydrogel” have higher frequency, therefore, these keywords can represent the main research topics in the field of biomaterials. Except for these, most of the keywords are closely connected to each other, which reveals that the main studies in the field of biomaterials were interlinked.

#### Emerging trends and frontiers

**Figure 7:**
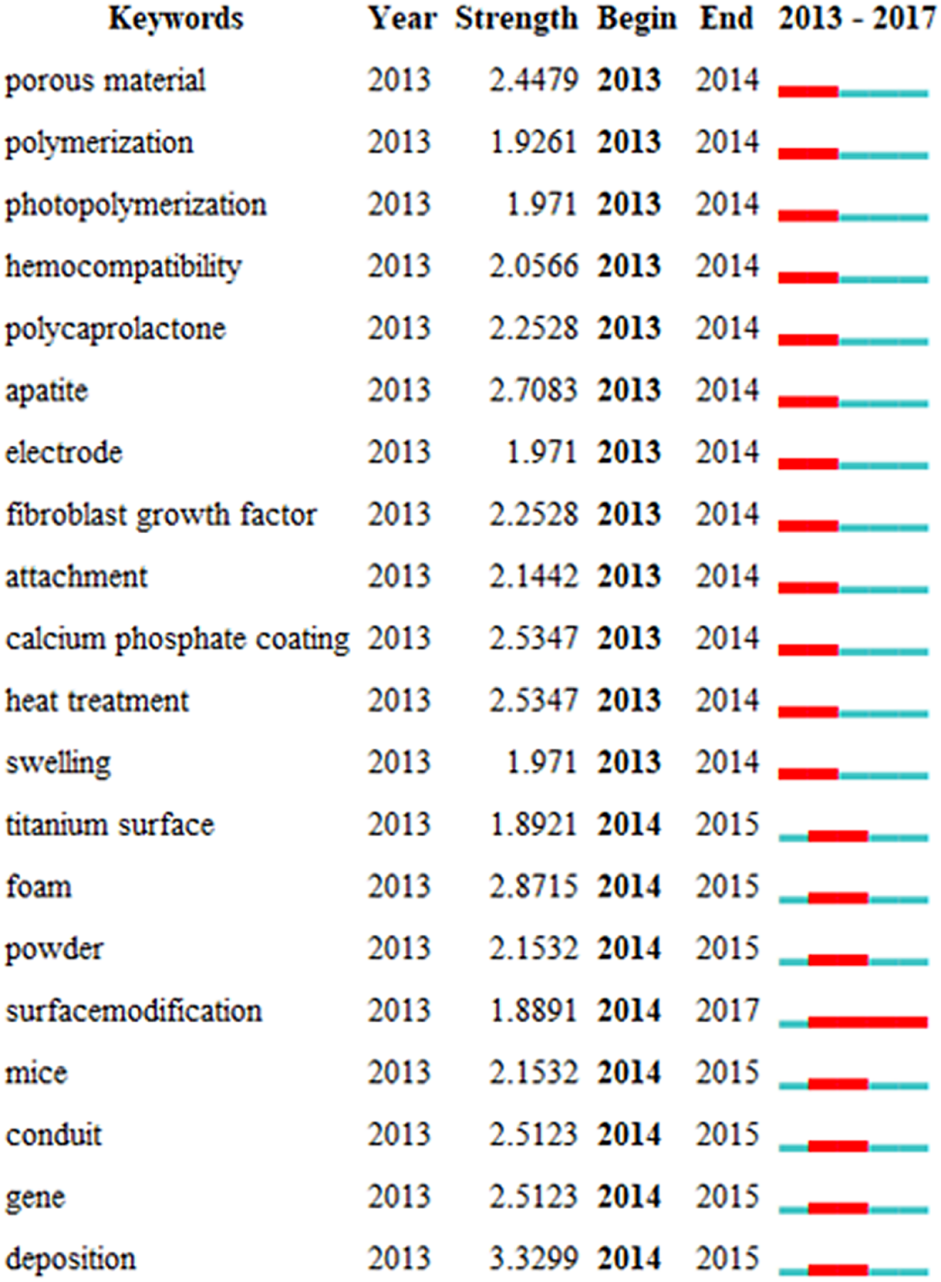
Top 20 keywords with the strongest citation burst. The top 20 keywords with the strongest citation burst were analyzed. The keywords with a strong citation burst usually represent the emerging trend of a specific research.

The keywords with a strong citation burst usually represent the emerging trends of a specific research (Zhou et al., 2018; Qiu & Liu, 2018). Here, we analyze the burst keywords to investigate the emerging trends in the field of biomaterials. The top 20 keywords with the strongest citation burst were analyzed, as shown in Fig. 7. The burst keywords are different in different time. From 2013 to 2014, the keywords “apatite”, “heat treatment”, “calcium phosphate coating” and “porous material” have stronger burst strength value; from 2014 to 2015, the keywords “deposition”, “foam” have the strongest citation burst, and the keyword “surface modification” is the only word with the burst duration until 2017. It is reasonable to speculate that studies related to these keywords can be considered as the emerging trends in the field of biomaterials during the different time. Furthermore, the keyword “surface modification” can represent a new active topic, and even the major frontier in the field of biomaterials, because its burst duration has lasted until 2017. Therefore, research related to this keyword may unceasingly affect the development of biomaterials.

**Table 2:** List of authors with frequency ≥30. The nine high-frequency authors with frequency ≥30 are shown.

Serial number	Frequency	Centrality	Name
1	51	0.23	Wang L
2	47	0.11	Liu Y
3	44	0.19	Zhang J
4	41	0.17	Zhang Y
5	40	0.09	Wang Y
6	38	0.11	Li Y
7	34	0.18	Wang H
8	34	0.13	Wang J
9	30	0.04	Chen Y

## Discussion

In this study, the general status and evolutions of research contents of articles in the biomaterials of China were investigated by using bibliometric analysis, ORIGIN, and CiteSpace software, based on the literature collected from the Web of Science Core Collection. Here, we limit the time to 2013–2017 because biomaterials in China had been developed most vigorously and importantly for future development based on how the government had energetically increased investment in biomaterials research, and issued a many vital policies to provide guidance for the development of biomaterials during this period. Despite this limitation, we believe that the results of this study can also be used as a reference for biomaterials development of not only China but also all other countries, because China is a large country producing articles in this field.

Biomaterials are the basis for the study of artificial organs and medical devices, and have become a hot spot of research and development by scientists all over the world. In this article, whether in terms of quantity, quality of articles or research teams in the field of biomaterials all show that there are growing concerns about the development of biomaterials in China. The in vitro test as an economical and time-saving method, which first established in 1926 (Hanks, Wataha & Sun, 1996), has been used to predict biological reactions when biomaterials were placed into the body. The high frequency keywords, “in vitro” and “scaffold” demonstrate that the detection of biomaterials has integrated the in vitro biocompatibility test with in vivo evaluation, indicating that the application of biomaterials has gradually become the focus of researchers. The fabrication of biomaterials mainly focuses on the third generation of bioactive materials, which endow biomaterials with unique performance characteristics and biological activities, thereby regulating and manipulating human proteins and cells to achieve the repair and regeneration of tissues and organs. These can be summarized as the relationship between the three-dimensional environment constructed by biomaterials and the physiological environment required for cells proliferation and differentiation and even tissue regeneration. It is worth noting that the growing need is what incited researchers pay more attention to the biomaterials’ manufacture and application. As far as we know, bio-regenerative materials have a large demand market. Drug materials for cancer treatment have also become the most important demand direction, including drug controlled release carriers, nanomaterials and so on. In addition, the surface modification technology of biomaterials has become one of the important means to meet various needs. Driven by the huge market demand, more creations in the field of biomaterials will be made.

## Conclusion

In terms of the quality and quantity of published articles, the research level of China in the field of biomaterials has been greatly improved. China already has strong teams in this research field. The keywords “*in vitro*”, “scaffold”, “nanoparticle”, “mechanical property”, “biocompatibility”, “drug delivery”, and “surface” have been the research hotspots during the period of 2013 to 2017, while the keyword “surface modification” may be the frontier in this field, and the researchers should pay attention to the related studies in the future.

##  Supplemental Information

10.7717/peerj.6859/supp-1 Dataset S1 The number of different type papers in every year in the field of biomaterials of China from 2013 to 2017

10.7717/peerj.6859/supp-2 Dataset S2 Journals of the biomaterials published in 2013

10.7717/peerj.6859/supp-3 Dataset S3 Journals of the biomaterials published in 2014

10.7717/peerj.6859/supp-4 Dataset S4 Journals of the biomaterials published in 2015

10.7717/peerj.6859/supp-5 Dataset S5 Journals of the biomaterials published in 2016

10.7717/peerj.6859/supp-6 Dataset S6 Journals of the biomaterials published in 2017

10.7717/peerj.6859/supp-7 Dataset S7 Articles in different quartiles of 2013

10.7717/peerj.6859/supp-8 Dataset S8 Articles in different quartiles in 2014

10.7717/peerj.6859/supp-9 Dataset S9 Articles in different quartiles of 2015

10.7717/peerj.6859/supp-10 Dataset S10 Articles in different quartiles of 2016

10.7717/peerj.6859/supp-11 Dataset S11 Articles in different quartiles of 2017

## Additional Information and Declarations

### Data Availability

The following information was supplied regarding data availability: The raw data are available in Datasets S1–S11.
